# Construction of a novel kinetic model for the production process of a CVA6 VLP vaccine in CHO cells

**DOI:** 10.1007/s10616-023-00598-8

**Published:** 2023-10-27

**Authors:** Zhou Xing, Thao Bich Nguyen, Guirong Kanai-Bai, Noriko Yamano-Adachi, Takeshi Omasa

**Affiliations:** 1 Graduate School of Engineering, Osaka University, U1E801, 2-1Yamadaoka, Suita, Osaka 565-0871 Japan; 2 Institute for Open and Transdisciplinary Research Initiatives, U1E801, 2-1 Yamadaoka, Suita, Osaka 565-0871 Japan; 3 Present Address: Tsukuba Research Laboratories, Eisai Co. Ltd, 5-1-3 Tokodai, Tsukuba, Ibaraki 300-2635 Japan

**Keywords:** Vaccine production, Virus-like particle, Chinese hamster ovary cell culture, Bioprocess modeling, Process optimization

## Abstract

Bioprocess development benefits from kinetic models in many aspects, including scale-up, optimization, and process understanding. However, current models are unable to simulate the production process of a coxsackievirus A6 (CVA6) virus-like particle (VLP) vaccine using Chinese hamster ovary cell culture. In this study, a novel kinetic model was constructed, correlating (1) cell growth, death, and lysis kinetics, (2) metabolism of major metabolites, and (3) CVA6 VLP production. To construct the model, two batches of a laboratory-scale 2 L bioreactor cell culture were prepared and various pH shift strategies were applied to examine the effect of pH shift. The proposed model described the experimental data under various conditions with high accuracy and quantified the effect of pH shift. Next, cell culture performance with various pH shift timings was predicted by the calibrated model. A trade-off relationship was found between product yield and quality. Consequently, multiple objective optimization was performed by integrating desirability methodology with model simulation. Finally, the optimal operating conditions that balanced product yield and quality were predicted. In general, the proposed model improved the process understanding and enabled in silico process development of a CVA6 VLP vaccine.

**Supplementary Information:**

The online version contains supplementary material available at 10.1007/s10616-023-00598-8.

## Introduction

Hand, foot, and mouth disease (HFMD) is a viral illness that usually occurs in children. In most cases, the disease is mild and patients recover spontaneously within several days. However, severe complications may occur in some patients, which can even lead to death. HFMD has had many outbreaks worldwide (Mirand et al. 2021; Puenpa et al. 2019; Solomon et al. 2010; Wu et al. 2010), posing a threat to public health.

HFMD is caused by enteroviruses which are non-enveloped viruses with various serotypes. Coxsackievirus A6 (CVA6), which is an enterovirus serotype, is gaining attention because of its increasing incidence during this decade (Kimmis et al. 2018). CVA6 can cause adult HFMD (Ramirez-Fort et al. 2014), and no CVA6 vaccine is available.

A virus-like particle (VLP) vaccine is a novel type of vaccine. It triggers humoral and cellular immune responses by resembling the protein coat of a specific virus (Fuenmayor et al. 2017). Compared with conventional inactivated and live-attenuated vaccines, VLP vaccines are safer because genetic material is absent in the VLPs (Nooraei et al. 2021). Several VLP-based vaccines have been approved by the Food and Drug Administration, becoming commercially available, while others are undergoing clinical trials (Nooraei et al. 2021). Therefore, a CVA6 VLP vaccine is a promising candidate prevention measure for HFMD.

Several expression systems are available for VLP production, including bacterial, yeast, insect, plant, and mammalian cells (Fuenmayor et al. 2017). Among these platforms, Chinese hamster ovary (CHO) cells were selected to produce CVA6 VLP vaccine in this study because of several advantages. CHO cells are able to grow in chemically defined and serum-free medium, ensuring batch consistency of the product and reducing the burden of downstream processing (Bandaranayake and Almo 2014). Chemically defined serum-free medium is also desired from a regulatory standpoint because no unknown materials are contained (Lai et al. 2013). These cells can also be adapted to suspension culture, which guarantees scalability for large-scale industrial production. Furthermore, the majority of human viruses cannot replicate in CHO cells, which reduces the biosafety risk (Bandaranayake and Almo 2014; Lai et al. 2013). CHO cells can also produce proteins with complex human-like post-translational modifications (O’Flaherty et al. 2020).

To produce a high-quality CVA6 VLP vaccine with high efficiency and consistency, the production process needs to be developed iteratively. Kinetic modeling, which is derived from physical, chemical, and biological parameters governing the process, is a powerful and versatile tool in the production of biopharmaceuticals because it enables thoughtful use and quantitative analysis and prediction of experimental data (Shirsat et al. 2015a, b). For example, kinetic models can be used as a filter to remove measurement errors and systematic noise, and add missing data points, so that high-quality data of the bioprocess are generated (Zhang et al. 2019). Additionally, kinetic models have been applied to aid online monitoring and control of bioprocesses (Hille et al. 2020; Krämer and King 2016). This application is encouraged by the regulatory agency through Process Analytical Technology (PAT) initiatives, promoting quantitative tools for real-time quality assurance (Narayanan et al. 2019). Process optimization and decision-making also benefit from kinetic modeling. A kinetic model has been combined with the design of experiment (DoE) methodology for multi-objective optimization of an antibody production process (Möller et al. 2019). This model-assisted method significantly reduced the number of experiments required compared with the traditional design of experiment and therefore reduced the cost and time. Moreover, kinetics-based models facilitate bioprocess scale-up (Arndt et al. 2021) and improve the understanding of the production process (Luo et al. 2021). Despite these benefits, no kinetic model is available for the VLP production process. Many kinetic models for recombinant protein production using CHO cell culture have been established (Kyriakopoulos et al. 2018). In these mechanistic models, the product titer is commonly correlated to viable cells using a parameter termed specific productivity. However, for non-enveloped VLP production, cell lysis is required to release the intracellular VLPs (Cervera and Kamen 2018). Similarly, we observed that VLP concentration in the supernatant was very low when viable cell density reached its peak. By contrast, production of VLPs mostly occurred during the dead phase (after day 8) and a strong correlation (R^2^ > 0.95) were found between the VLP concentration and the sum of dead cell concentration and lysed cell concentration, instead of viable cells. Therefore, well-established models of recombinant protein production are inapplicable to the VLP vaccine production process. Therefore, we proposed a newly developed kinetic model for the VLP vaccine production process to enhance the process understanding, enable in silico optimization, and perform quantitative decision making.

In this study, a mathematical model that describes process dynamics was constructed to simulate fed-batch cultivation of CVA6 VLP-producing CHO-S cells using laboratory-scale bioreactors. Cell cultures were conducted with a pH shift on day 10 and without a pH shift to determine the effects of pH on the CVA6 VLP titer. After calibrating the model, sensitivity analysis was carried out to quantify the effect of model parameters. Then, culture performance with various pH shift timings was predicted to optimize the VLP yield. Finally, multiple objective optimization balancing product yield and quality was conducted by combining model simulation and desirability methodology.

## Materials and methods

### Cell line, medium, and preculture

A patented CVA6-VLP-producing CHO-S cell line (Kuwabara et al. 2020) provided by BIKEN Group Japan (Osaka, Japan) was cultivated in CD FortiCHO™ medium (Thermo Fisher Scientific, Waltham, MA, USA) supplemented with 8 mM glutamine (Fujifilm Wako, Osaka, Japan). Cell cultures using 0.5 L Optimum Growth® Flasks (Thomson Instrument, CA, USA) with 0.1 L working volume were performed after cell thawing and expansion. Cell cultivation was performed in a humidified incubator (Climo-Shaker, Kuhner, Switzerland) operated at 37 °C, 8% CO_2_, and 140 rpm. Cells were subcultured every 3–4 days when they were during the exponential phase prior to fed-batch bioreactor cultivation.

### Fed-batch laboratory-scale bioreactor cultivation

Laboratory-scale bioreactor cell culture was performed using a stirred tank glass bioreactor (ABLE Biott, Tokyo, Japan) with a 2 L maximum working volume operated in fed-batch mode. Inoculation was performed by seeding cells at 4 × 10^8^ cells/L in a 0.8 L working volume. Cultivation conditions were controlled at 30% dissolved oxygen using a pure O_2_ sparge, 80 rpm agitation, 0.1 L/min constant air flow, and 8 × 10^–3^ L/min constant CO_2_ flow. Temperature was controlled at 37 °C and then changed to 32 °C after day 5. pH maintenance was conducted by sparging CO_2_ gas and 1 M NaHCO_3_ addition. In the control experiment (bioreactor 1), pH was maintained at 7.15 until the end of culture. In the treatment experiment (bioreactor 2), pH was maintained at 7.15 and then changed to 6.75 after day 10.

Up to 3 × 10^–3^ L FoamAway™ (Thermo Fisher Scientific) was added to when foaming was observed during cell culture. From day 3, 1.6 × 10^–2^ L of feed medium (EfficientFeed™ C + AGT™ Supplement, Thermo Fisher Scientific), which was equal to 2% of the initial volume, was fed into the bioreactor every day. Additional glucose supplementation was conducted by adding a 300 g/L glucose stock solution to ensure a glucose concentration of > 2 g/L. Then, 4 × 10^–3^ L of sample was collected from the bioreactor daily for cell growth, CVA6 VLP concentration, and metabolite analyses.

### Cell counting and metabolite measurements

Viable cell concentration and viability were measured by a ViCell automated cell counter (Beckman Coulter, Brea, CA, USA). Metabolite (glucose, glutamine, lactate, and ammonia) concentrations were quantified using a BioProfile 400 Automated Chemistry Analyzer (Nova Biomedical, Waltham, MA, USA).

### Cell lysis quantification

Cell lysis quantification was performed by adapting a previously proposed method that uses double-stranded DNA (dsDNA) as an indicator (Klein et al. 2015). Briefly, dsDNA in supernatant samples was measured using a Quant-iT PicoGreen dsDNA Assay Kit (Thermo Fisher Scientific). Supernatant samples were diluted appropriately and 1 × 10^5^ μg/L Lambda DNA Standard was diluted to prepare the standard curve. Samples and the standard were mixed with assay reagent in a 96-well plate. After incubation at room temperature for 2 min, a VICTOR Nivo Multimode Microplate Reader (PerkinElmer, Waltham, MA, USA) was used to excite samples and the standard at 492 nm, and fluorescence emission intensities at 530 nm were measured. Intracellular dsDNA content was determined by the mass balance under the assumption that cell growth stopped after the exponential phase (after day 5). By dividing dsDNA in the supernatant by intracellular dsDNA content, the lysed cell density was quantified. The spontaneous release of dsDNA from viable cells was neglected because dsDNA concentration in the supernatant during the exponential phase was negligible compared with that during the dead phase.

### CVA6-VLP quantification

A sandwich enzyme-linked immunosorbent assay developed by Biken Group Japan was applied for CVA6 VLP quantification. Briefly, 96-well plates were coated with an anti-CVA6 monoclonal antibody and incubated at 4 °C overnight. Non-specific binding was blocked by incubation with EzBlock Chemi (Atto, Tokyo, Japan) blocking buffer for 1 h at 37 °C. Then, microplates were washed thrice with washing buffer (0.05% (v/v) Tween 20 in phosphate-buffered saline) prior to addition of the standard or samples. Microplates were incubated at 37 °C for 1 h, followed by three washes. A horseradish peroxidase-conjugated anti-CVA6 monoclonal antibody was added as the secondary antibody. After 1 h of incubation at 37 °C, microplates were washed three times and binding was visualized by incubation with a TMB solution (Surmodics, MN, USA) for 15 min. To stop the reaction, BioFX 650 nm Liquid Stop Solution for TMB Microwell substrates (Surmodics) was added to each well. Absorbance at 650 nm was read by a microplate reader (Corona Electrics, Ibaraki, Japan).

### Model construction

Kinetics between cells, major metabolites, and VLP were described by ordinary differential equations. Volume changes during fed-batch cultivation were disregarded because they were negligible compared with the working volume.

1-a $$\frac{{dX_{t} }}{dt} = rX_{t}$$

1-b $$r = r_{max} \cdot \left( {1 - \frac{{X_{t} }}{{X_{t,max} }}} \right)$$

where ${X}_{t}$ denotes total cell density; $r$ denotes intrinsic growth rate and ${r}_{max}$ denotes maximum intrinsic growth rate; ${X}_{t,max}$ denotes maximum total cell density.

A logistic equation (Martínez et al. 2020; Shirsat et al. 2015a, b) was applied to describe the behavior of the total cell density (${X}_{t}$) to prevent overfitting because it had fewer parameters than Monod-type equations. This equation also complied with the previous assumption that cell proliferation stopped during the late stage of culture.

2-a $$\frac{{dX_{d} }}{dt} = k_{D} X_{v} - k_{DL} X_{d}$$

If (${c}_{Amm}$>${c}_{Amm,cr}$ during dead phase):

2-b $$k_{D} = k_{D, dead} \cdot \frac{{K_{{D_{Amm} }} + c_{Amm} - c_{Amm,cr} }}{{K_{{D_{Amm} }} }}$$

Else:

2-c $$k_{D} = k_{D, min}$$

3 $$\frac{{dX_{l} }}{dt} = k_{L} X_{v} + k_{DL} X_{d}$$

4 $$\frac{{dX_{v} }}{dt} = rX_{t} - \left( {k_{D} + k_{L} } \right) \cdot X_{v}$$

where ${X}_{d}$ is dead cell density and ${X}_{v}$ is viable cell density; ${k}_{D}$ is specific death rate and ${k}_{DL}$ is specific lysis rate from dead cells; ${k}_{D, dead}$ stands for death rate during dead phase; ${K}_{{D}_{Amm}}$ stands for constant for cell death due to ammonia accumulation, ${c}_{Amm}$ stands for ammonia concentration and ${c}_{Amm,cr}$ stands for critical ammonia concentration for specific death rate; ${k}_{D, min}$ is minimum death rate and ${k}_{L}$ is specific lysis rate from viable cells; ${X}_{l}$ denotes lysed cell density.

**Fig. 1:**
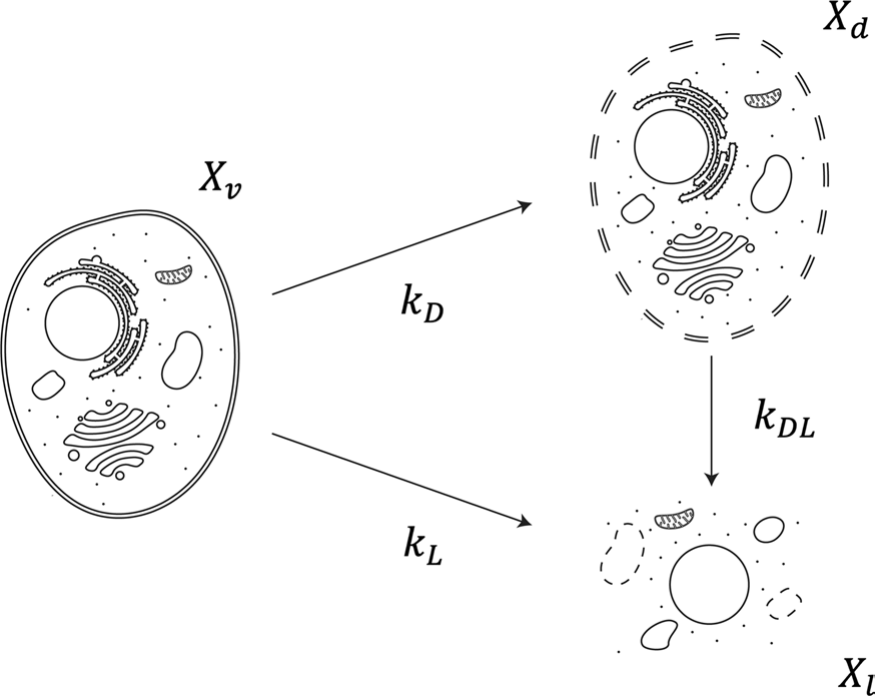
Cell death and lysis pathways with their corresponding rate coefficients. Adapted from Kroll et al. (2017). This figure was created by Adobe Illustrator 2023 (Adobe Inc., CA, USA)

Cell death and lysis pathways as well as their corresponding coefficients are illustrated in Fig. 1. These pathways were modeled by adapting previously proposed equations (Kontoravdi et al. 2007; Kroll et al. 2017). Briefly, cell death was accelerated significantly when the ammonia concentration exceeded its critical concentration (${c}_{Amm,cr}$ = 5 mM) during the dead phase (Kontoravdi et al. 2007). Additionally, lysed cells (${X}_{l}$) could be generated by viable cells (${X}_{v}$) directly or dead cells (${X}_{d}$) as shown by the two terms in Eq. (3), respectively. ${X}_{v}$ was the difference between ${X}_{t}$ and the sum of ${X}_{d}$ and ${X}_{l}$.

5 $$\frac{{dc_{Glc} }}{dt} = - \left( {rX_{t} - \left( {k_{D} + k_{L} } \right)X_{v} } \right)/Y_{{X_{v} /Glc}} - m_{Glc} X_{v} + \frac{{V_{F,i} c_{M} }}{V} + \frac{{V_{G,i} c_{G} }}{V}$$

Before day 5:

6-a $$\frac{{dc_{Lac} }}{dt} = \left( {\left( {rX_{t} - \left( {k_{D} + k_{L} } \right) \cdot X_{v} } \right)/Y_{{X_{v} /Glc}} - m_{Glc} X_{v} } \right) \cdot Y_{Lac/Glc}$$

After day 5:

6-b $$\frac{{dc_{Lac} }}{dt} = \left( {\left( {rX_{t} - \left( {k_{D} + k_{L} } \right) \cdot X_{v} } \right)/Y_{{X_{v} /Glc}} - m_{Glc} X_{v} } \right) \cdot Y_{Lac/Glc} - q_{Lac} X_{v}$$

6-c $$q_{Lac} = q_{Lac,max} \cdot \frac{{c_{Lac} }}{{K_{Lac} + c_{Lac} }}$$

where ${c}_{Glc}$ and ${c}_{Lac}$ are glucose and lactate concentrations, respectively; ${Y}_{{X}_{v}/Glc}$ is yield coefficient of cell proliferation to glucose uptake; ${V}_{F,i}$ and ${V}_{G,i}$ denote the volumes of feed medium and glucose supplementation at $i$ th feeding, respectively; ${c}_{M}$ and ${c}_{G}$ are the glucose concentrations of the feed medium and glucose supplementation, and $V$ is the working volume; ${m}_{Glc}$ is specific glucose consumption rate for cell maintenance; ${Y}_{Lac/Glc}$ represents yield coefficient of lactate production to glucose uptake and ${q}_{Lac}$ represents specific lactate uptake rate; ${q}_{Lac,max}$ is maximum lactate uptake rate and ${K}_{Lac}$ is Monod kinetic constant for lactate uptake.

**Fig. 2:**
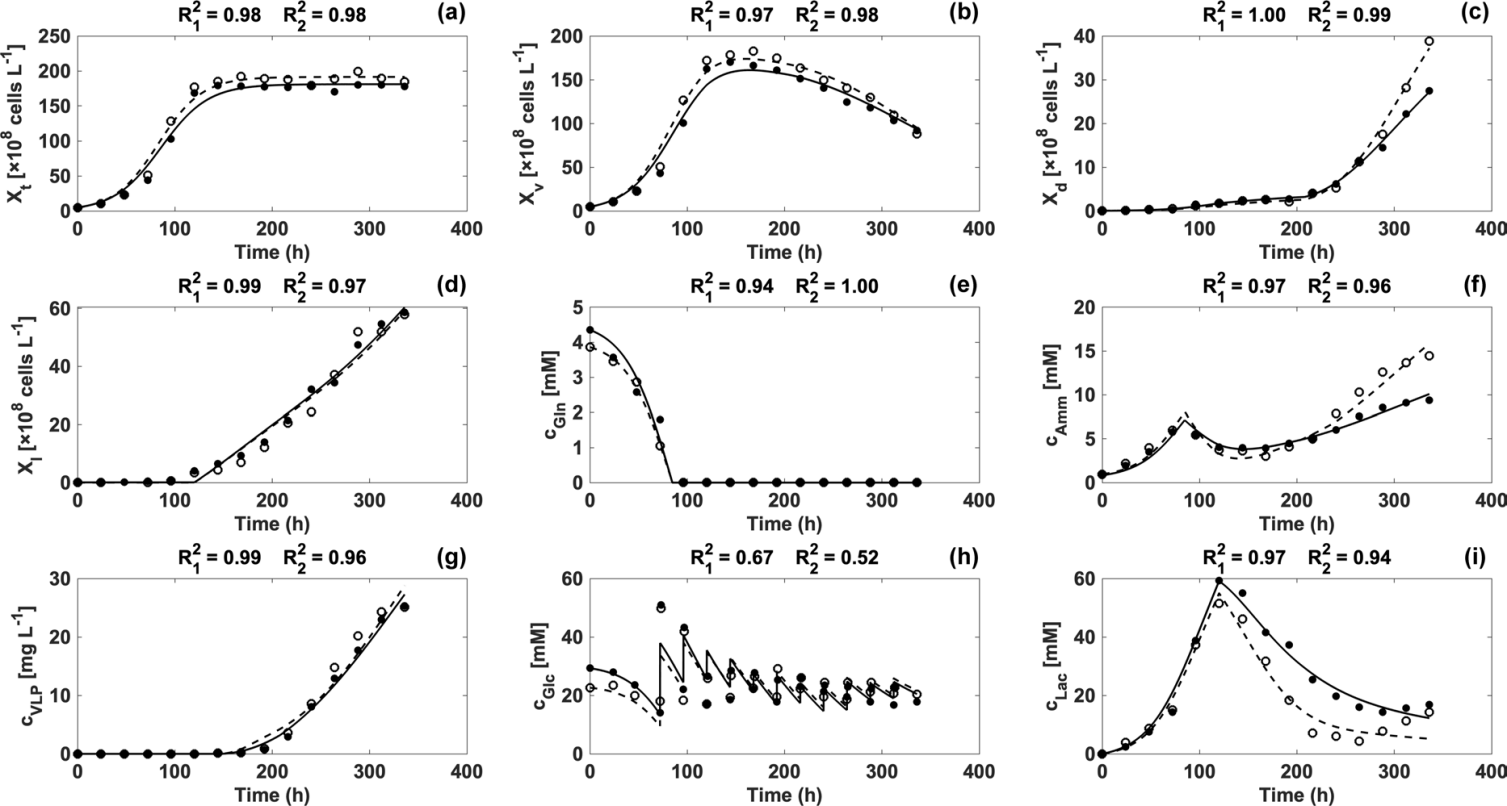
Comparison between model simulations of bioreactor 1 (solid line) and bioreactor 2 (dash line) (Edward 2022) versus experimental data of bioreactor 1 (closed circle) and bioreactor 2 (open circle) for nine variables (a–i). R^2^ was used to evaluate the goodness of fit. This figure was created by MATLAB R2021a (The Math Works, Inc., MA, USA)

Equation (5) was modified from a study by Xing et al. which consists of glucose consumption due to cell growth and maintenance as well as the increase in glucose concentration as a result of feeding (Xing et al. 2010). The change of lactate concentration was modeled by Eq. (6). Cells switched from lactate production to lactate consumption after day 5, which can be observed in Fig. 2i. The mechanism of this kind of lactate switch remains unclear, although it is common in CHO cell lines (Hartley et al. 2018). A yield coefficient (${Y}_{Lac/Glc}$) was used to link glucose use and lactate production. After day 5, an additional Monod-type term was applied to account for lactate consumption.

If ($${c}_{Gln}$$ > 0 mM):

7-a $$\frac{{dc_{Gln} }}{dt} = - \left( {rX_{t} - \left( {k_{D} + k_{DL} } \right) \cdot X_{v} } \right)/Y_{{X_{v} /Gln}}$$

8-a $$\frac{{dc_{Amm} }}{dt} = \left( {rX_{t} - \left( {k_{D} + k_{DL} } \right) \cdot X_{v} } \right)/Y_{{X_{v} /G\ln }} \cdot Y_{Amm/G\ln }$$

If ($${c}_{Gln}$$ = 0 mM):

7-b $$\frac{{dc_{Gln} }}{dt} = 0$$

8-b $$\frac{{dc_{Amm} }}{dt} = - \left( {rX_{t} - \left( {k_{D} + k_{DL} } \right) \cdot X_{v} } \right)/Y_{{X_{v} /Amm}} + q_{Amm} X_{v}$$

where ${c}_{Gln}$ and ${c}_{Amm}$ denote glutamine and ammonia concentrations, respectively; ${Y}_{{X}_{v}/Gln}$ denotes yield coefficient of cell proliferation to glutamine uptake and ${Y}_{Amm/Gln}$ denotes yield coefficient of ammonia production to glutamine uptake; ${Y}_{{X}_{v}/Amm}$ yield coefficient of cell proliferation to ammonia uptake and ${q}_{Amm}$ is specific ammonia production rate.

Similarly, glutamine consumption was described by a yield coefficient and ammonia was considered to be the production of glutamine when glutamine was available. When glutamine was depleted, ammonia was used as an alternative nitrogen source and consumed until the end of the cell growth phase. This phenomenon has also been reported in other mammalian cell lines (Lie et al. 2019). Additionally, ammonia production for cell maintenance was modeled through ${q}_{Amm}$, which differs at various pHs. After day 6. If (${VLP}_{intra}<{VLP}_{max}$ and pH = 7.15):

9-a $$\frac{{dVLP_{intra} }}{dt} = q_{VLP}$$

If (${VLP}_{intra}={VLP}_{max}$ or pH = 6.75):

9-b $$\frac{{dVLP_{intra} }}{dt} = 0$$

10 $$\frac{{dc_{VLP} }}{dt} = VLP_{intra} \cdot \left( {k_{D} + k_{L} } \right) \cdot X_{v}$$

where ${VLP}_{intra}$ is the amount of VLPs accumulated intracellularly, and ${q}_{VLP}$ is its accumulation rate; ${c}_{VLP}$ denotes VLP concentration in the medium.

Our previous observations indicated that the VLPs were produced efficiently after day 6. Moreover, a pH downshift suppressed production of intracellular VLPs. Additionally, there was a maximal intracellular VLP content that a single cell can contain (${VLP}_{max}$). The VLP concentration in the medium was simulated by combining ${VLP}_{intra}$ with cell release, either cell death or lysis, as shown by Eq. (10).

The computational work was conducted in MATLAB R2021a and Optimization Toolbox (The Math Works, Inc., MA, USA). Ordinary differential equations were solved by ode45 solver in MATLAB. Parameters were determined by minimizing weighted root-mean-square deviation between the experimental data and model simulation. Model simulation was evaluated by the coefficient of determination (R^2^) (Colin Cameron and Windmeijer 1997).

### Local sensitivity analysis

To further understand the effect of various model coefficients and initial culture conditions, local sensitivity analysis was carried out. Briefly, the relative change in the final VLP concentration by the end of cell culture was simulated in response to a 10% change in model parameters independently. Model parameters of bioreactors 1 and 2 before day 10 were averaged because two bioreactors were operated under the same conditions before the pH shift on day 10. Coefficients after the pH shift were based on the simulation of bioreactor 2. Local sensitivity was simulated under the same pH shift strategy in bioreactor 2.

### Predictions under various pH shift timings

After model calibration, determined parameters were used to predict the experimental performance of the VLP production process under various pH shift strategies. Various time-dependent cell densities, major metabolite concentrations, and VLP yield were predicted to understand and optimize the process of VLP vaccine production.

### Multiple objective optimization

Desirability methodology was applied to optimize the production process with multiple responses (${y}_{i}$) such as metabolite concentrations or the VLP concentration. In brief, various responses were used to compute an individual desirability function (${d}_{i}\left({y}_{i}\right)$), which was either maximization (Eq. 11-a) or minimization (Eq. 11-b) depending on customized objectives (Möller et al. 2019). Individual desirability ${d}_{i}\left({y}_{i}\right)$ ranged from 0 to 1. The objective was satisfied if its ${d}_{i}\left({y}_{i}\right)$ was close to 1. The overall desirability was obtained by multiplication of individual objectives as illustrated by Eq. (12), which was used as a quantitative criterion for comparison to guide decision making.

11-a $$d_{i} \left( {y_{i} } \right) = \left\{ {\begin{array}{*{20}l} 0 \hfill & {{\text{if}}{\mkern 1mu} y_{i} < L_{i} } \hfill \\ {\left( {\frac{{y_{i} - L_{i} }}{{U_{i} - L_{i} }}} \right)} \hfill & {{\text{if}}{\mkern 1mu} L_{i} < y_{i} < U_{i} } \hfill \\ 1 \hfill & {{\text{if}}{\mkern 1mu} y_{i} < U_{i} } \hfill \\ \end{array} } \right.$$

11-b $$d_{i} \left( {y_{i} } \right) = \left\{ {\begin{array}{*{20}c} {1\quad {\text{if}}\,y_{i} < L_{i} } \\ {\left( {\frac{{U_{i} - y_{i} }}{{U_{i} - L_{i} }}} \right)\quad {\text{if}}\,L_{i} < y_{i} < U_{i} } \\ {0\quad {\text{if}}\,y_{i} > U_{i} } \\ \end{array} } \right.$$

12 $$D = { }\mathop \prod \limits_{{{\text{i }} = { }1}}^{{\text{n}}} d_{i} \left( {y_{i} } \right) = { }d_{1} \left( {y_{1} } \right) \times d_{2} \left( {y_{2} } \right) \cdots \times d_{n} \left( {y_{n} } \right)$$

where ${U}_{i}$ and ${L}_{i}$ are customized upper and lower boundaries, respectively. By integrating the designed desirability function with the model simulation, multiple objective optimization was performed to predict the optimal pH shift strategy and timing to stop the batch.

## Results and discussion

### Simulation of bioreactor cultivation

Parameters in the model equations were estimated to fit two batches of bioreactor cell culture by the aforementioned parameter determination method. A comparison between experimental data and model simulation is shown in Fig. 2. Additionally, some model parameters are listed in Table 1. As shown in Fig. 2a, simulation of the total cell densities of the two bioreactors fitted the experimental data with high accuracy. The two batches of bioreactor culture had similar profiles because the two bioreactors were operated under the same conditions during the growth phase. Model parameters related to total cell density (${r}_{max}$ and ${X}_{t,max}$) were also comparable between batches.

**Table 1:** Comparison of model parameters between bioreactors 1 and 2

Parameter	Bioreactor 1	Bioreactor 2
${r}_{max}$ (h^−1^)	0.042	0.044
${X}_{t, max}$ (10^8^ cells L^−1^)	181.1	191.5
${k}_{D, min}$ (10^–4^ h^−1^)	2.5	1.7
${k}_{DL, 32}$ (10^–3^ h^−1^)	8.8	6.9
${k}_{L, 32}$ (10^–3^ h^−1^)	1.4	1.3
${k}_{D,dead}$ (10^–4^ h^−1^)	5.1	3.3
${K}_{{D}_{Amm}}$ (mM)	0.59	0.60
${Y}_{{X}_{v/Gln}}$ (10^12^ cells mol^−1^)	1.90	2.45
${Y}_{Amm/Gln}$ (−)	1.45	1.84
${Y}_{{X}_{v/Amm}}$ (10^12^ cells mol^−1^)	1.74	1.08
${q}_{Amm, 7.15}$ (10^–15^ mol cell^−1^ h^−1^)	0.95	1.65
${q}_{Amm, 6.75}$ (10^–15^ mol cell^−1^ h^−1^)	N.A	2.66
${VLP}_{max}$ (10^–12^ g cell^−1^)	3.85	3.39
${q}_{VLP}$ (10^–13^ g cell^−1^ h^−1^)	0.59	1.08
${t}_{VLP,cr}$ (h)	209.5	175.3

In addition to the total cell density, this model thoroughly described viable cell densities (Fig. 2b) and dead cell densities (Fig. 2c) with R^2^ > 0.95. Compared with bioreactor 1, cell death in the late stage of cell culture was increased in bioreactor 2 after the pH shift. The phenomenon was caused by accelerated accumulation of ammonia due to the pH downshift (Fig. 2f). As shown in Fig. 2f, ammonia concentrations (${c}_{Amm}$) were estimated by the proposed model. At the beginning, ammonia was produced by glutamine consumption. When glutamine was depleted, ammonia started to be consumed to support cell growth until the viable cell density reached its peak. Other mammalian cell lines have also been reported to exhibit similar behavior (Lie et al. 2019). Subsequently, the ammonia concentration increased again because of cell maintenance (Xu et al. 2019). After a pH shift to 6.75, the ammonia production rate (${q}_{Amm,6.75}$) was increased significantly compared with the specific ammonia production rate at pH 7.15 (${q}_{Amm,7.15}$), resulting in a higher ammonia level than in the control bioreactor, which was consistent with the observation of Lee et al. (2021). As a result, cell death was accelerated in bioreactor 2. Overall, the model successfully described ammonia concentrations during cell culture, despite small deviations between data points and simulation in the final stage. These deviations were mainly caused by the simplicity of the model. Additionally, the effect of the pH shift was quantified successfully through the change in ${q}_{Amm}$.

In terms of the lysed cell density (Fig. 2d), the kinetic model provided precise descriptions of the data. Model simplicity and experimental error contributed to model deviation. Data from the two bioreactors did not show significant differences, and model parameters related to cell lysis and specific cell lysis rates from dead and viable cells at 32 °C (${k}_{DL,32}$ and ${k}_{L,32}$) were comparable, which suggested that the effect of the pH shift on cell lysis was negligible. Additionally, glutamine consumption was modeled accurately (Fig. 2e). The difference in the initial glutamine concentrations might be due to experimental and measurement errors.

Figure 2g shows the experimental data and model simulation of the VLP concentration (${c}_{VLP}$) over the cultivation time. VLP production was effectively simulated by the proposed model. After day 6, VLPs started to be released into the medium. VLP concentrations in two bioreactors had similar profiles, although there were more dead cells in bioreactor 2 because of the higher ammonia concentration due to the pH shift. The reason was that every single cell in bioreactor 2 contained fewer intracellular VLPs on average. ${t}_{VLP, cr}$ indicates the critical time for intracellular VLP accumulation to reach maximum intracellular VLP content (${VLP}_{max}$). As shown in Table 1, ${t}_{VLP, cr}$ was shorter than 240 h in each bioreactor, which means that intracellular VLP accumulation achieved its maximum and stopped before the pH shift. Therefore, VLP concentration was the product of the sum of dead and lysed cell density and ${VLP}_{max}$. Even though there were more dead and lysed cells in bioreactor 2, ${VLP}_{max}$ was lower in bioreactor 2 (3.85 $\times$ 10^–12^ g cells^−1^ in bioreactor 1 and 3.39 $\times$ 10^–12^ g cells^−1^ in bioreactor 2) indicating that every single cell contained fewer VLPs on average. As a result, the VLP concentration was not improved much in bioreactor 2. In addition, lower ${VLP}_{max}$ was due to the batch-to-batch variation which was probably caused by the nature of cells because ${VLP}_{max}$ was reached before the pH shift. Two bioreactors were operated under the same conditions before pH was shifted on day 10. Besides, because the newly proposed equation correlating cell death and lysis with VLP concentration (Eq. 10) is able to describe CVA6 VLP concentration during the cell culture precisely, it provides an opportunity of modeling production process of other products which are produced intracellularly such as other non-enveloped VLPs. More concretely, according to our observations, the production of Enterovirus 71, Coxsackievirus A10, Coxsackievirus A16, and Norovirus VLPs, which are non-enveloped VLPs, has shown similar behavior to the CVA6 VLP. Their product concentrations were also strongly correlated to dead and lysed cell densities. Such kind of behavior can be modeled by Eq. 10 as well. Besides, this kinetic model can also be modified to model other non-enveloped VLP vaccine production process since the product release of other non-enveloped VLP relies on cell lysis as well (Cervera and Kamen 2018). To customize the model to other production processes, equations of cell growth, death and lysis can be modified since the limiting substrate can be different in various cells. Similar to Eq. 10, product concentration can be correlated to dead and lysed cell densities subsequently. Simulating dead and lysed cell densities accurately is critical for modeling product concentration, as these densities can affect product concentration directly.

As shown in Fig. 2h, the kinetic model only provided an estimation of glucose concentrations in two bioreactors with low accuracy of R^2^ = 0.67 and 0.52, respectively. The main reason for the low accuracy was the simplicity of the model. Only two model parameters were used to describe the behavior of glucose concentration. Fewer parameters were helpful for the generality by preventing overfitting. Increasing the number of model parameters would improve the model accuracy. For example, the specific glucose consumption rate could be changed from a constant to a Monod-type term. However, it is noteworthy that there exists a trade-off between generality and precision in the field of modeling mammalian cells (Shirsat et al. 2015a, b). In this case, the precision of glucose concentration simulation was sacrificed because glucose was kept sufficient and did not affect the product yield. In addition, experimental and measurement errors also contributed to the significant deviation between experimental data and model simulation. For instance, addition of feeding medium as well as glucose supplement could introduce human error. Simulation of the lactate concentration is shown in Fig. 2i. Lactate was produced during the exponential growth phase and switched to consumption after day 5. The dynamic model described the lactate concentration with reasonable accuracy.

### Local sensitivity analysis

**Fig. 3:**
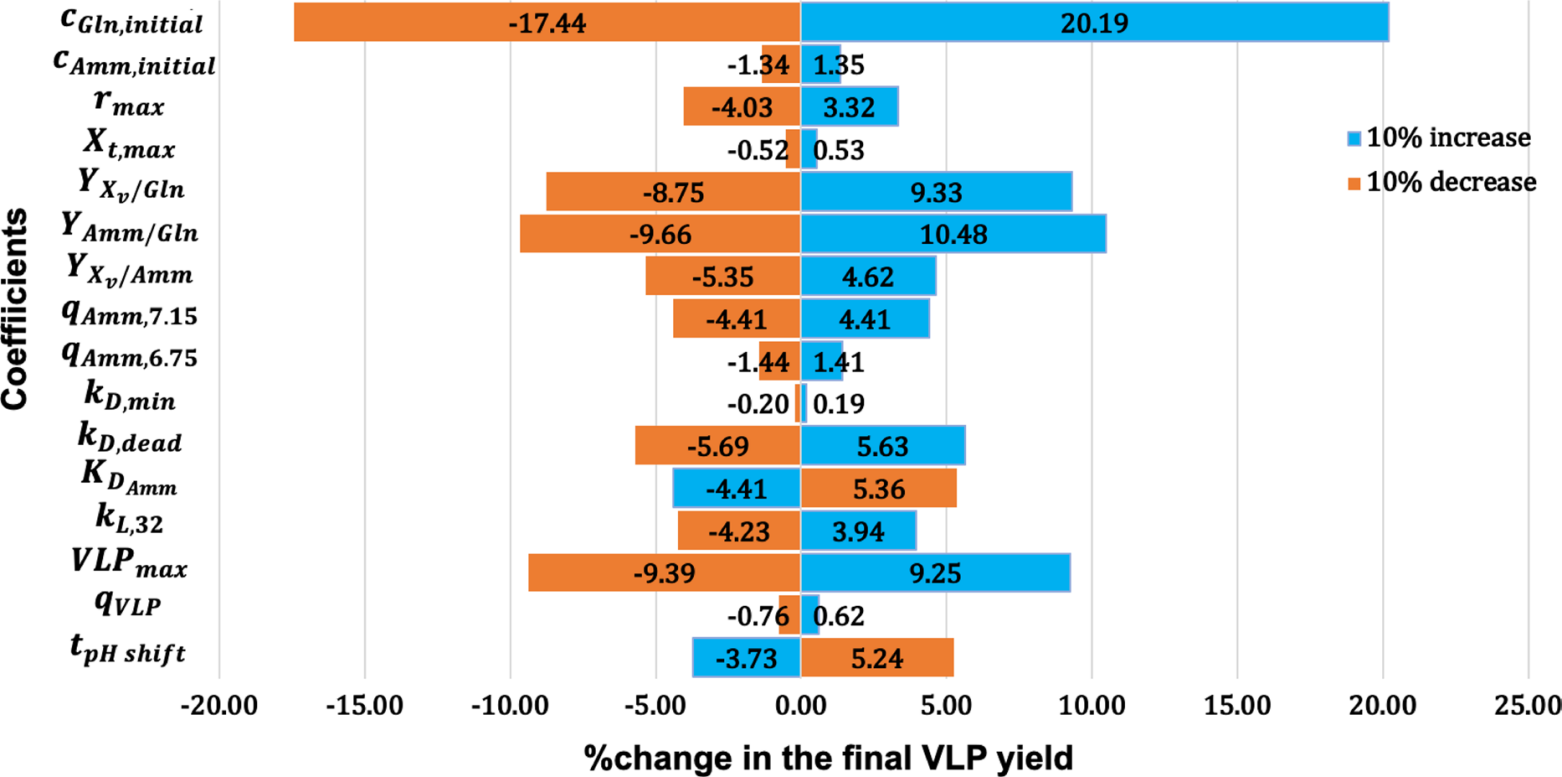
Results of local sensitivity analysis. This figure was created by Microsoft® PowerPoint 2022 Version 16.61 (Microsoft Corporation, WA, USA)

Batch-to-batch variation existed between the two bioreactors as suggested by the variance in model parameters and initial conditions. The change in the final VLP yield in response to the variance in model parameters and initial conditions needed to be examined. Therefore, local sensitivity analysis was carried out to determine the effect of the coefficients used in the proposed model on the final product yield. The analysis was performed in a one-way manner, which changed one factor by 10% while keeping other factors constant to observe the fractional change in the output (Qian and Mahdi 2020). A tornado plot was used to display the results of sensitivity analysis (Fig. 3). Ammonia was the metabolite related to cell death. Additionally, glutamine consumption was correlated to ammonia production in the kinetic model. Therefore, the effect of variance in the initial concentrations of ammonia and glutamine (${c}_{Amm,initial}$ and ${c}_{Gln,initial}$) on the final VLP yield was investigated. Glutamine supplementation in the medium was critical for VLP production because a 10% increase in the initial glutamine concentration increased the final VLP concentration by 20.19%. The increase in the glutamine concentration led to a higher level of ammonia, which increased the number of dead cells. Consequently, the release of VLPs was enhanced. Conversely, changes in the initial ammonia concentration were unable to cause significant differences in the final product yield because the numerical value of ${c}_{Amm,initial}$ was too low. In summary, the sensitivity analysis of initial conditions showed that glutamine supplementation was an important material attribute affected the product yield, whereas the final VLP yield was resistant to the variance in the ammonia concentration at the beginning.

Fractional changes in model parameters also affected VLP production. The decrease in ${r}_{max}$ had a negative effect on the VLP yield because it delayed cell growth, and therefore reduced the numbers of dead and lysed cells. Similarly, the reduction in ${X}_{t, max}$ was also adverse for VLP production, but it only had a limited effect. Yield coefficients also considerably changed the final VLP concentration because they influenced the ammonia level during cultivation and subsequently affected cell death. Specifically, if ${Y}_{{X}_{v}/Gln}$, ${Y}_{Amm/Gln}$, or ${Y}_{{X}_{v}/Amm}$ was increased by 10% independently, the final VLP amount would be changed by 9.33%, 10.48%, and 4.62%, respectively. Additionally, ${q}_{Amm,7.15}$ and ${q}_{Amm,6.75}$ played roles in the product concentration because they indirectly affected cell death by influencing the ammonia concentration. Increases in these coefficients accelerated the accumulation of ammonia, which increased cell death and eventually promoted the release of VLPs. Moreover, variances in model parameters related to cell death and lysis induced changes in the product yield. ${K}_{D,min}$ only had a minimal effect on the final VLP yield because it was a parameter during the early stage of cell culture, whereas cell death mostly occurred during the late stage. However, 10% changes in ${k}_{D,dead}$, ${K}_{{D}_{Amm}}$, and ${k}_{L,32}$ led to a 4–6% difference in the final product concentration. In terms of parameters directly related to VLP production, ${VLP}_{max}$ had a considerable effect because a 10% decrease reduced the VLP yield by 9.39%. However, ${q}_{VLP}$ only had a limited effect. Production of VLPs was also affected by the pH shift timing. The results of local sensitivity analysis suggested the potential of optimizing the VLP production process by shifting the pH earlier. It would be beneficial to perform additional experiments to validate the simulation results of sensitivity analysis. However, in this case, model validation was difficult to perform. For instance, it was challenging to increase specific death rate by 10% independently without changing other process parameters.

### Effect of the pH shift timing on VLP production

**Fig. 4:**
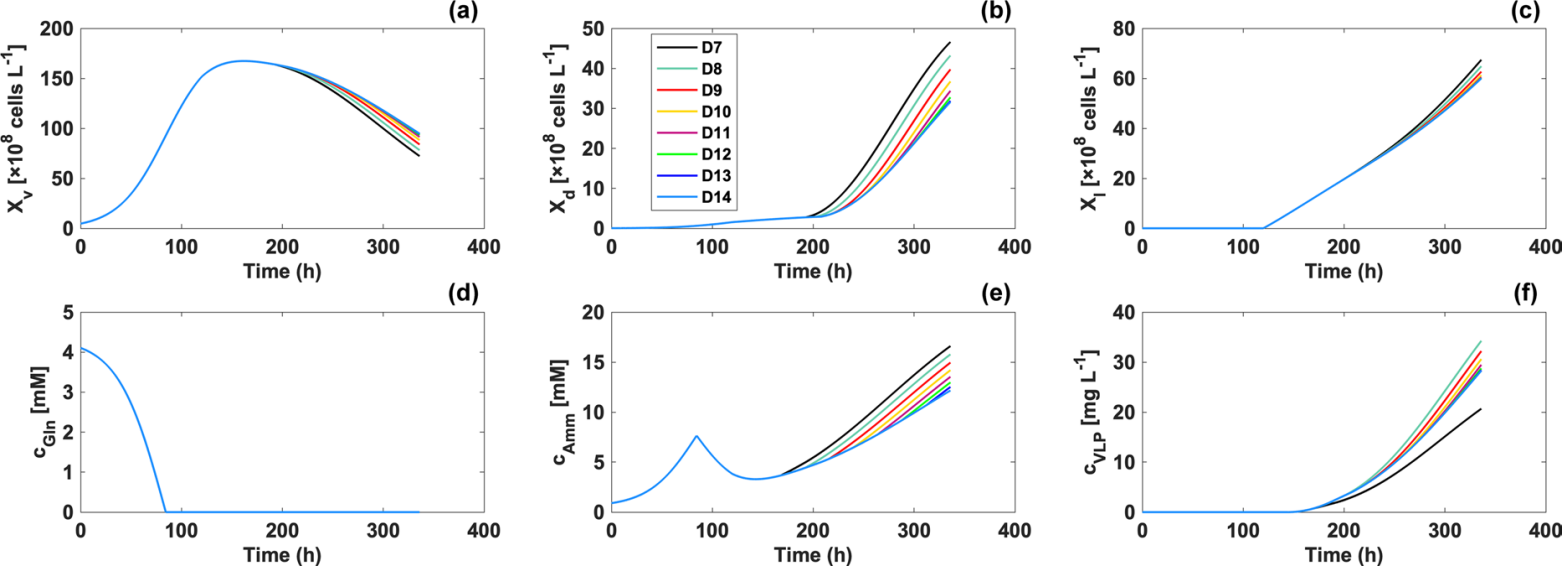
Prediction of experimental performance of the VLP vaccine production process with various pH shift timings for six variables (a–h). D8–14 denote the day when a pH shift is performed. This figure was created by MATLAB R2021a (The Math Works, Inc., MA, USA)

To further investigate experimental performance under various pH shift timings, a prediction was performed using determined model coefficients (Fig. 4). For the simulation, model parameters and initial conditions were averaged when applicable. D8 to D14 denote the pH downshift timing from day 8 to 14 (without a pH shift). On the basis of the course of ${c}_{Amm}$ predicted in Fig. 4e, ammonia accumulation was accelerated after the pH shift. Consequently, ${X}_{v}$ decreased faster under strategies in which pH was shifted on earlier days. Furthermore, if the pH was downshifted on early days, ${X}_{d}$ (Fig. 4b) and ${X}_{l}$ (Fig. 4c) tended to be higher by the end of cultivation. As shown in Fig. 4d, the pH shift timing hardly affected glutamine consumption. The time-dependent course of VLP concentrations was also predicted. Despite the highest number of dead and lysed cells, a pH shift on day 7 resulted in the lowest yield, which was due to the low ${VLP}_{intra}$ because the pH downshift stopped the accumulation of ${VLP}_{intra}$, and ${VLP}_{max}$ could not be reached. If pH was shifted on day 8 or later days, ${VLP}_{max}$ was achievable. When ${VLP}_{intra}$ reached${VLP}_{max}$, the higher ${X}_{d}$ and ${X}_{l}$ released more VLPs. Therefore, except for day 7, the final VLP yield was decreasing as the pH shift timing was later. The highest yield was achieved by shifting the pH on day 8, which generated an approximately 20% increase in the final product concentration compared with the cell culture without a pH shift. However, the presence of batch-to-batch variation posed difficulty for the validation of prediction results. For example, as mentioned before, after the pH shift, there were more dead and lysed cells in bioreactor 2. Thus, it was supposed to be higher yield in bioreactor 2. Nevertheless, the product concentration was not improved much because of the batch-to-batch variation of cells. Even though model validation was absent, simulation results were in line with previous reports. More specifically, elevated ammonia level due to the pH downshift was also reported by several studies (Lee et al. 2021; Trummer et al. 2006). Considering ammonia was reported to be toxic for cells (Schneider 1996) and be able to induce cellular apoptosis (Wang et al. 2018), it was logical that the model predicted there would be more dead cells if pH was shifted earlier because of higher ammonia concentration.

### Multiple objective optimization

In the field of biopharmaceutical production, maximizing product yield is not the only criterion. Product quality also needs to be guaranteed. Our results showed that a pH shift increased the ammonia level at the late stage of cell culture, which accelerated cell death and consequently resulted in a higher yield. Nevertheless, a high ammonia concentration also has a negative effect on glycosylation gene expression in CHO cells resulting in inhibited glycosylation of recombinant proteins (Chen and Harcum 2006). VLPs are also a type of recombinant proteins and previous studies have demonstrated that glycosylation is critical for triggering the immune response as well as the function and structure of VLPs, such as glycoprotein folding and VLP assembly (Chen and Lai 2013; Lavado‐García et al. 2022). Thus, there is a trade-off between product yield and quality. To enable quantitative decision making of the trade-off, the model simulation was integrated with desirability methodology. A desirability function was designed to maximize the VLP concentration while simultaneously minimizing the ammonia concentration.

**Fig. 5:**
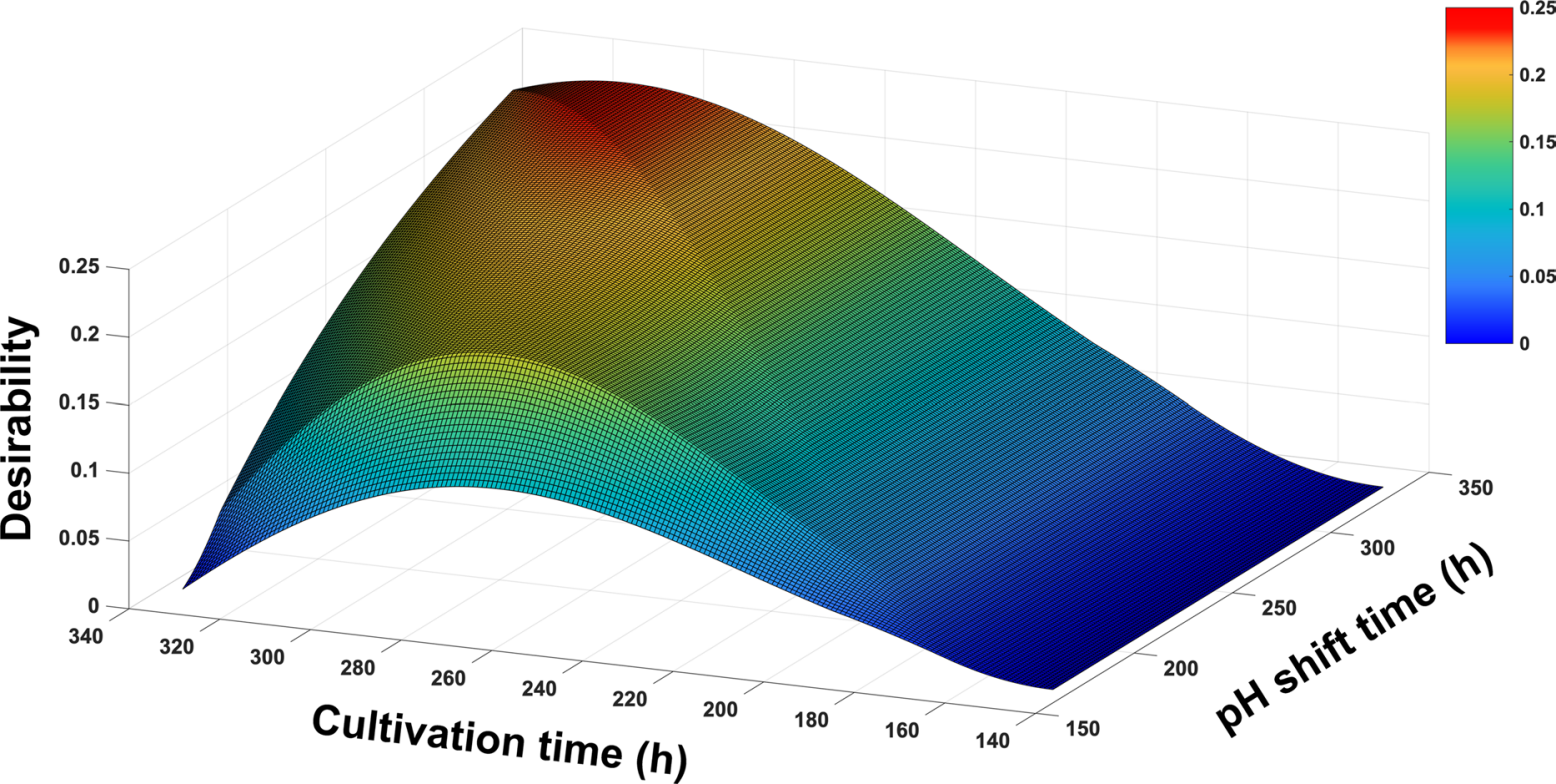
Surface plot of desirability as a function of cultivation and pH shift timings. This figure was created by MATLAB R2021a (The Math Works, Inc., MA, USA)

The result was visualized by a surface plot (Fig. 5). In general, as the cell culture proceeded, the value of desirability increased at the beginning because the VLP concentration kept increasing during this period. Nevertheless, desirability decreased during the late stage because of the high ammonia concentration. Additionally, the delay in the pH shift timing had a positive effect on the overall desirability. Notably, desirability did not change with respect to the change in pH shift timing if the pH shift timing was later than the cultivation timing, which was indicated by the diagonal on the surface. Cell culture without a pH shift at 311 h was predicted to be the most desired operating condition that balanced the product yield and quality.

## Conclusion

In this study, a kinetic model was constructed for the VLP vaccine production process using CHO cell culture to fulfill the gap of currently available models which cannot simulate the pattern of VLP production. The constructed model simulated the experimental data from two batches of bioreactor cell cultures under various pH shift strategies with high accuracy. It also revealed the mechanism of cellular behaviors with respect to metabolite concentrations. The newly proposed equation correlating VLP concentration with cell death and lysis provides an opportunity of modeling other non-enveloped VLP production process. The determined model parameters enabled quantitative comparison between the two bioreactors, so that the effects of a pH shift was quantified and provided a better understanding of the process dynamics. By performing sensitivity analysis, glutamine supplementation in the medium was suggested to be an important material attribute that considerably affected the final VLP yield. Therefore, to guarantee efficient VLP vaccine production, glutamine supplementation has to be designed and controlled carefully. Subsequently, the calibrated model was used to predict the performance of the VLP vaccine production process if a pH shift was conducted on various days. Day 8 was the optimal day to shift the pH to achieve the highest VLP yield. However, there was a trade-off between product yield and quality. To balance them, desirability methodology was integrated with the model simulation to perform multiple objective optimization. Cell culture without a pH shift at 311 h was predicted to be the most desired operating strategy, which balanced the product yield and quality. Hence, the use of this model enabled quantitative decision making for process optimization in silico, so that the cost and time to conduct experiments were reduced. In conclusion, the newly proposed model is a versatile tool to facilitate development of the VLP vaccine production process. It also has the potential to support hybrid modeling, which integrates a mechanistic model and data-driven methods such as artificial neural network (Narayanan et al. 2019). Additionally, both mechanistic and hybrid modeling play important roles in digital twin, which enables process development in silico and is desired by the biopharmaceutical industry as bioprocesses are moving towards Industry 4.0 (Cardillo et al. 2021; von Stosch et al. 2021). Cell culture performance has been suggested to be different even under the same conditions. Therefore, further research may develop stochastic models that use distribution to quantify batch-to-batch variation because batch consistency is also important for biopharmaceutical production.

### Supplementary Information

Below is the link to the electronic supplementary material.

Supplementary file1 (XLSX 15 KB)

## **Abbreviations**

CHO: Chinese hamster ovary
DoE: Design of experiment
dsDNA: Double-stranded DNA
HFMD: Hand, foot and mouth disease
PAT: Process analytical technology
VLP: Virus-like particle

## **List of symbols**

*c*_*Amm*_: Ammonia concentration (mM)
${c}_{Amm,cr}$: Critical ammonia concentration for specific death rate (mM)
${c}_{G}$: Glucose concentration in the glucose supplement (mM)
${c}_{Glc}$: Glucose concentration (mM)
${c}_{Gln}$: Glutamine concentration (mM)
${c}_{Lac}$: Lactate concentration (mM)
${c}_{M}$: Glucose concentration in the feed medium (mM)
${k}_{D}$: Specific death rate (h^−^^1^)
${k}_{D, dead}$: Death rate during dead phase (h^−^^1^)
${k}_{D, min}$: Minimum death rate (h^−^^1^)
${k}_{DL}$: Specific lysis rate from dead cells (h^−^^1^)
${k}_{DL,32}$: Specific lysis rate from dead cells at 32 °C (h^−^^1^)
${k}_{L}$: Specific lysis rate from viable cells (h^−^^1^)
${k}_{L,32}$: Specific lysis rate from viable cells at 32 °C (h^−^^1^)
${K}_{{D}_{Amm}}$: Constant for cell death due to ammonia accumulation (mM)
${K}_{Lac}$: Monod kinetic constant for lactate uptake (mM)
${m}_{Glc}$: Specific glucose consumption rate for cell maintenance (mol cell^−^^1^ h^−^^1^)
${q}_{Amm}$: Specific ammonia production rate (mol cell^−^^1^ h^−^^1^)
${q}_{Amm,6.75}$: Specific ammonia production rate at pH of 6.75 (mol cell^−^^1^ h^−^^1^)
${q}_{Amm,7.15}$: Specific ammonia production rate at pH of 7.15 (mol cell^−^^1^ h^−^^1^)
${q}_{Lac}$: Specific lactate uptake rate (mol cell^−^^1^ h^−^^1^)
${q}_{Lac,max}$: Maximum lactate uptake rate (mol cell^−^^1^ h^−^^1^)
${q}_{VLP}$: Specific intracellular VLP accumulation rate (g cell^−^^1^ h^−^^1^)
$r$: Intrinsic growth rate (h^−^^1^)
${r}_{max}$: Maximum intrinsic growth rate (h^−^^1^)
t: Time (h)
$$V$$: Working volume (L)
${V}_{F,i}$: Volume of feed medium at $i$ th feeding (L)
${V}_{G,i}$: Volume of glucose supplement at $i$ th feeding (L)
${VLP}_{intra}$: Intracellular VLP content (g cell^−^^1^)
${VLP}_{max}$: Maximum intracellular VLP content (g cell^−^^1^)
${X}_{d}$: Dead cell density (cells L^−^^1^)
${X}_{l}$: Lysed cell density (cells L^−^^1^)
${X}_{t}$: Total cell density (cells L^−^^1^)
${X}_{t,max}$: Maximum total cell density (cells L^−^^1^)
${X}_{v}$: Viable cell density (cells L^−^^1^)
${Y}_{Amm/Gln}$: Yield coefficient of ammonia production to glutamine uptake (-)
${Y}_{Lac/Glc}$: Yield coefficient of lactate production to glucose uptake (-)
${Y}_{{X}_{v}/Amm}$: Yield coefficient of cell proliferation to ammonia uptake (cells mol^−^^1^)
${Y}_{{X}_{v}/Glc}$: Yield coefficient of cell proliferation to glucose uptake (cells mol^−^^1^)
${Y}_{{X}_{v}/Gln}$: Yield coefficient of cell proliferation to glutamine uptake (cells mol^−^^1^)
